# Understanding sleep and smartphone use in diverse adolescents through passive digital monitoring

**DOI:** 10.1111/jora.70192

**Published:** 2026-05-06

**Authors:** Oren Shahnovsky, Daniella Ekstein, Talia Friedman, Maya Stemmer, Lior Carmi, Noa Benaroya‐Milshtein, Shai Fine, Shira Barzilay

**Affiliations:** ^1^ Department of Community Mental Health, Faculty of Social Welfare and Health Sciences University of Haifa Haifa Israel; ^2^ Department of Child and Adolescent Psychiatry Schneider Children's Medical Center of Israel Petah Tikvah Israel; ^3^ Data Science Institute Reichman University Herzliya Israel; ^4^ Gray Faculty of Medical & Health Sciences Tel Aviv University Tel‐Aviv Israel

**Keywords:** adolescents, minority populations, passive sensing, passive smartphone monitoring, suicide

## Abstract

Adolescents are among the most frequent smartphone users worldwide. Yet, few studies have examined how smartphone use appears among minority adolescents, including sexual and gender minority (SGM) youth and children of immigrant parents, who often experience unique stressors and heightened mental‐health risk. Passive smartphone monitoring provides a promising, low‐burden method for continuously and objectively assessing real‐world behavior, offering new opportunities to identify dynamic markers of mental health challenges, including suicide risk, in daily life. The present study evaluated the feasibility of a replicable framework for passive smartphone monitoring among adolescents at high risk for suicidal thoughts and behaviors (STB) and explored longitudinal differences in smartphone‐derived behavioral features across minority subgroups. Ninety‐nine adolescents aged 11–18 with recent STB completed baseline assessments and installed the *iFeel* app, which collected passive smartphone data for 6 months, including total and social‐media screen time and phone‐inactivity‐based proxy sleep indicators inferred from nighttime phone inactivity. Participants contributed 1500 participant‐weeks of data, with an average of 11.9 weeks of valid monitoring, supporting the feasibility and acceptability of this approach. Daily smartphone use time, social‐media activity time, and sleep duration were comparable to normative adolescent data. No significant longitudinal differences emerged between SGM and non‐SGM adolescents. However, immigrant‐origin adolescents displayed shorter but more stable sleep patterns compared to non‐immigrant origins, who exhibited longer baseline sleep with steeper declines over time. Findings highlight passive sensing as a feasible, inclusive, and scalable method for examining real‐world behavioral processes associated with STB and mental health outcomes among diverse adolescents. This framework offers a scalable approach that future studies can apply to deepen real‐time understanding of mental‐health challenges and behavioral patterns among diverse adolescents.

## ADOLESCENT SMARTPHONE ENGAGEMENT

There are more than 7 billion smartphone users worldwide, a number expected to reach nearly 8 billion by 2028 (Taylor, 2024). Adolescents stand out as one of the most active groups, spending significantly more time using their smartphones compared to previous years (Camerini et al., 2021). Recent studies show that adolescents average at least 5 h of daily phone use, with many continuing to be online well into the night (Alexander et al., 2024). This high level of engagement is particularly concerning, given the critical neurodevelopmental changes of the adolescent brain, which makes them more susceptible to potential negative effects of excessive smartphone use (Erus et al., 2015).

In a recent study, distinct trajectories of addictive social media use, phone usage, and gaming were identified from childhood to early adolescence, with addictive patterns linked to suicidal thoughts and behaviors (STB) and poor mental health outcomes. Nearly half of the participants followed a trajectory of highly addictive mobile phone use, and more than 40 percent exhibited similar patterns for video games. By the end of 4 years, nearly one in three adolescents developed high levels of addictive social media use, and one in four reached similar levels for phone use (Xiao et al., 2025).

While such findings highlight the general risks associated with addictive digital media use, they also raise important questions about which adolescents may be most vulnerable. Evidence suggests that demographic and contextual factors can shape patterns of smartphone use and related technologies (Wang et al., 2025). For example, boys are more likely to use smartphones for gaming and browsing, whereas girls tend to use their devices for social interaction (González‐Bueso et al., 2018).

## SMARTPHONE USE PATTERNS AND SLEEP IN MINORITY GROUPS

### Sexual and gender minority (SGM)

Minority adolescents, specifically sexual and gender minorities (SGM), may be disproportionately affected by problematic smartphone use. For SGM adolescents, social media platforms provide critical tools for social connection and identity expression in contexts marked by stigma or lack of acceptance (Gudelunas, 2012; Hillier & Harrison, 2007; Huang et al., 2025). At the same time, reliance on smartphones in this population has been linked to reduced perceived social support and heightened symptoms of depression and anxiety (Vogel et al., 2021).

Recent research reveals that SGM adolescents report significantly more screen time than heterosexual peers, including higher levels of mobile phone use, video gaming, and social media addiction (Kaiser et al., 2021; Nagata et al., 2023). Adolescents who were uncertain about their SGM status also reported elevated problematic use. These patterns suggest that SGM adolescents are drawn into intensive media use, raising concerns about potential consequences for overall mental health.

Sleep may represent a key mechanism in this association. Minority adolescents are disproportionately affected by chronic sleep deprivation and sleep‐related disorders, conditions consistently associated with adverse mental health outcomes, including STB (Bagley et al., 2015). SGM adolescents in particular report shorter sleep duration and poorer sleep quality compared to their heterosexual peers (Tepman & Wong, 2024), whereas better sleep quality appears protective in the context of minority stress (Nikolaidis‐Konstas et al., 2025). Importantly, smartphone use and problematic gaming are strongly associated with shorter sleep, increased daytime sleepiness, and a greater risk of sleep disorders (Kristensen et al., 2021; Narita et al., 2025). Together, these findings underscore the need to examine how smartphone use and sleep intersect to influence mental health in vulnerable populations.

### Immigrants

Immigrant adolescents experience elevated rates of psychological distress, depression, and anxiety compared to their non‐immigrant peers (Bas‐Sarmiento et al., 2017). Additionally, research has demonstrated that children of migrant origins often sleep less than their native‐born peers (Labree et al., 2015). However, research examining smartphone use and sleep patterns within this population remains limited. In particular, the relationship between smartphone use and sleep quality among immigrant adolescents has received little empirical attention. Given the established links between minority stress and maladaptive technology use observed in other minority groups (e.g., SGM), it is important to explore whether similar mechanisms operate among immigrant adolescents.

Background characteristics such as sexual and gender identity and immigrant origin may shape adolescents' daily behavioral routines, including patterns of smartphone engagement and sleep. For some adolescents, digital media may serve as a primary means of social connection, identity exploration, or coping with stress (Dreier et al., 2025), while sleep timing and regularity may be influenced by family structure, cultural norms, and environmental demands (Breitenstein et al., 2018). Among clinical adolescent populations, these background‐related factors may therefore be reflected in distinct longitudinal trajectories of passively measured behavior. These considerations highlight the need to investigate smartphone use and sleep quality within these minority populations to better understand differences in daily behavioral patterns across clinical adolescent samples.

## PASSIVE DIGITAL MONITORING AS AN UNTAPPED POTENTIAL

Despite a growing body of work on adolescent smartphone use, existing methods of measurement have notable limitations. Ecological Momentary Assessment (EMA) through self‐report surveys has provided valuable insight into the temporal dynamics of adolescent mental health (Czyz et al., 2022). However, it also introduces burden, fatigue, and missing data, particularly in adolescent samples (Jiang et al., 2023). Moreover, self‐reports are prone to recall errors and biases. For example, girls often underestimate their time on smartphones (Alexander et al., 2024), making accurate assessment difficult when based solely on adolescents' own reports. These limitations reveal a methodological gap within the field. Despite widespread use, current approaches may not adequately capture digital behavior in ecologically valid and low‐burden ways.

Passive digital monitoring provides a powerful means of overcoming these limitations by capturing behaviors and experiences in real‐time and within naturalistic settings (Alexander et al., 2024; Büscher et al., 2024; Jiang et al., 2023). This approach not only circumvents EMA fatigue and self‐report biases but also enables the identification of proximal predictors of mental health deterioration in real time, particularly among adolescents at elevated risk (Kleiman et al., 2023). In addition, passive sensing shows strong potential for equity, as it can extend participation to diverse adolescents who may face barriers to traditional care and research participation (Marciano & Saboor, 2023). Passive sensing enables low‐burden, continuous data collection through devices that adolescents already use in their daily lives (Camerini et al., 2021), capturing a more representative range of experiences. This approach not only enhances inclusivity but also improves ecological validity by reflecting real‐world contexts and behaviors. Feasibility studies confirm that adolescents can successfully participate in passive sensing protocols, highlighting their scalability and reliability (Huang et al., 2025).

## THE CURRENT STUDY

In this study, we build on evidence that smartphone usage patterns provide meaningful insights into daily routines (Auerbach et al., 2025). We propose that passive smartphone monitoring offers a scalable, ecologically valid tool for real‐time mental health assessment, particularly in underserved clinical populations. By passively capturing digital traces of activity, this method allows for a more nuanced understanding of how patterns of engagement unfold across time and context in adolescents' daily lives. Importantly, the unobtrusive nature of passive monitoring facilitates the inclusion of adolescents who are often underrepresented in research, such as SGM and immigrant‐origin adolescents. Notably, in the country where this research was conducted, both immigrant and SGM adolescents are minority groups and overrepresented in clinical samples at high risk for STB, highlighting the diversity of this study sample.

### Aims and hypotheses

The present study aimed to evaluate the feasibility and utility of passive smartphone monitoring in a high‐risk adolescent sample, and to examine longitudinal differences in smartphone‐derived behavioral features across key demographic subgroups.

Aim 1 To establish and evaluate a replicable framework for passive smartphone data collection, focusing on feasibility, data coverage, and quality. We hypothesize that passive data collection would be feasible, with most participants contributing usable data.
Aim 2 To examine longitudinal differences in smartphone‐derived behavioral features across demographic groups. Based on prior literature, we hypothesize that minority adolescents will demonstrate poorer sleep patterns and greater smartphone use across the study period compared to their non‐minority peers:
  Aim 2a Differences between sexual and gender minority (SGM) and non‐SGM adolescents.
  Aim 2b Differences between immigrant‐origin and non‐immigrant‐origin adolescents.

Through these aims, we seek to demonstrate the feasibility of implementing passive smartphone data collection in adolescent populations, while also advancing understanding of how behavioral features captured through these methods differ across minority and non‐minority adolescents over time.

## MATERIALS AND METHODS

### Study design

This study included 99 participants recruited from a specialized depression and self‐harm clinic at a large medical center. Participants were between the ages of 11 and 18 years, presenting to the emergency department with recent STB. Enrollment and data collection began in February 2021 and took place on a rolling basis, with the last participant being recruited in April 2023. Inclusion criteria were as follows: (a) history of STB; and (b) Android phone ownership (required for the iFeel app). Exclusion criteria included developmental or neurological disorders and intellectual disabilities that would prevent comprehension of research questions, as determined by a licensed psychiatrist or clinical psychologist.

### Procedure

Clinical staff notified the research team about potentially eligible participants. Senior research staff then met with families to explain the study in detail in accordance with local ethical requirements. Written parental consent and child assent (for participants aged 16 years and older) were obtained prior to enrollment. Participation or non‐participation in the study did not affect the standard psychosocial or pharmacological care provided by the clinic. After informed consent and assent were obtained, participants completed a comprehensive assessment battery and installed the iFeel app using a randomly generated code from the Google Play Store. The app collected smartphone‐based data for 6 months (25 weeks). To ensure consistent data flow and minimize technical disruptions, a trained research assistant monitored data collection weekly. Once a week, on a predetermined day (Tuesday) according to the clinic's working schedule, a dashboard was reviewed to verify that the app was active and transmitting information. In cases of missing data, connectivity issues, or app inactivity, the participant's family was contacted for troubleshooting purposes. This protocol ensured high‐quality passive sensing throughout the study period.

### Measures

#### Socio‐demographic characteristics

Socio‐demographic information was collected via parental report at intake, including immigration status, religious affiliation, socioeconomic status, and parental employment. Participants self‐reported gender identity and sexual orientation as part of the intake assessment.

Gender identity was assessed by asking participants to indicate whether they identified as male, female, transgender, or other. Sexual orientation was assessed separately, with response options including heterosexual, gay/lesbian, bisexual, or other. For analytic purposes, participants were classified as sexual and gender minority (SGM) if they identified as a gender minority and/or as a non‐heterosexual sexual orientation.

Regarding immigrant data, the study operationalized immigrant‐origin status based on parental country of birth: participants were classified as immigrant‐origin if at least one parent was born outside the country; otherwise, they were classified as non‐immigrant‐origin. The classification of “immigration” is complex and varies across studies (Stevens & Vollebergh, 2008). However, given the demographic characteristics and context of the country where this research was conducted, this approach was deemed the most appropriate to ensure the inclusion of minority participants.

#### Passive sensing screen use

The iFeel app continuously collected passive data 24 h a day, 7 days a week. For each participant, the app recorded the number of seconds spent in various applications. Applications were classified into 24 pre‐defined categories that were assigned automatically based on the Google Play Store taxonomy, including social media (e.g., TikTok, Instagram), gaming, browsers, and other application types. A full categorical list can be found in Appendix S1. These values were summed to generate a measure of total smartphone use (in seconds) for each participant. The data were originally recorded at an hourly resolution and were subsequently aggregated into weekly averages to reduce day‐to‐day variability and facilitate longitudinal modeling at a consistent temporal resolution. Smartphone use was further segmented into daytime and nighttime activity to capture temporal patterns of engagement. Based on these aggregated measures, we derived multiple phone‐inactivity‐based proxy sleep indicators. For the present study, the analyses focused on two primary indicators: total screen time and time spent on social media. For each category, the dataset captured total time spent (in seconds), separately calculated for day and night. All data were non‐identifiable and fully compliant with the General Data Protection Regulation (GDPR). No app‐specific content, messages, or sensitive information were collected.

#### Passive sensing sleep parameters

Total screen time during nighttime was used to infer sleep parameters. Following established behavioral assumptions that phone inactivity corresponds to sleep (Alexander et al., 2024), we derived five nightly phone‐inactivity‐based proxy sleep indicators. Screen inactivity was defined as periods with less than 60 s of total screen use. This threshold was selected to ensure that recorded activity reflected intentional engagement rather than background processes (e.g., app refreshes, notifications, or automated interactions). Although prior work has employed shorter durations to classify brief “checking behaviors” (Andrews et al., 2015), we adopted a more conservative 60‐s cutoff to improve accuracy in identifying sleep onset and wake time, thereby minimizing false positives. A night was considered valid if both sleep onset and wake time could be derived according to these inactivity criteria within the predefined nighttime and morning windows. Nights producing illogical sequences (e.g., wake time occurring before sleep onset) were excluded. A week was considered valid for analyses if it contained at least one valid night. Weekly sleep estimates were calculated as the mean of all valid nights within that week. From nighttime smartphone inactivity and activity patterns, we derived five phone‐inactivity‐based proxy sleep indicators (Ratzon et al., 2024):

1. *Sleep Onset Time*. Each hour was classified as “Nighttime” (9:00 PM–6:59 AM) or “Daytime” (7:00 AM–8:59 PM) based on phone activity. Sleep onset was defined as the first transition from wakefulness (screen use in the prior hour) to inactivity (no screen use in the current hour) within the nighttime interval.
2. *Wake‐Up Time*. Wake‐up time was estimated as the first hour between 6:00 AM and 11:00 AM in which a participant engaged in more than 60 s of screen activity. Only the earliest qualifying hour per day was retained as the wake‐up proxy.
3. *Sleep Duration*. Calculated as the interval (in hours) between the first sustained nighttime inactivity (sleep onset) and the first substantial screen activity the next morning (wake‐up). To accurately calculate sleep duration, it was necessary to account for the transition across midnight. We standardized the timeframe by adding 3 h to reported sleep onset times, thereby shifting the reference point beyond midnight. This adjustment ensured that all sleep periods spanning midnight could be consistently and accurately quantified.
4. *Sleep Disturbances (Awakenings)*. Defined as discrete instances of screen activation (exceeding 60 s) after sleep onset. For each participant, we counted the total number of such events and subtracted one to exclude the initial onset, thereby indexing nighttime awakenings.
5. *Wake After Sleep Onset (WASO)*. Estimated as the total duration of nighttime screen activity between 2:00 AM and 6:00 AM. This interval was selected because 88% of time points in the dataset were classified as sleep, supporting its validity as the primary sleep period in this sample.

### Implementation overview

**FIGURE 1:**
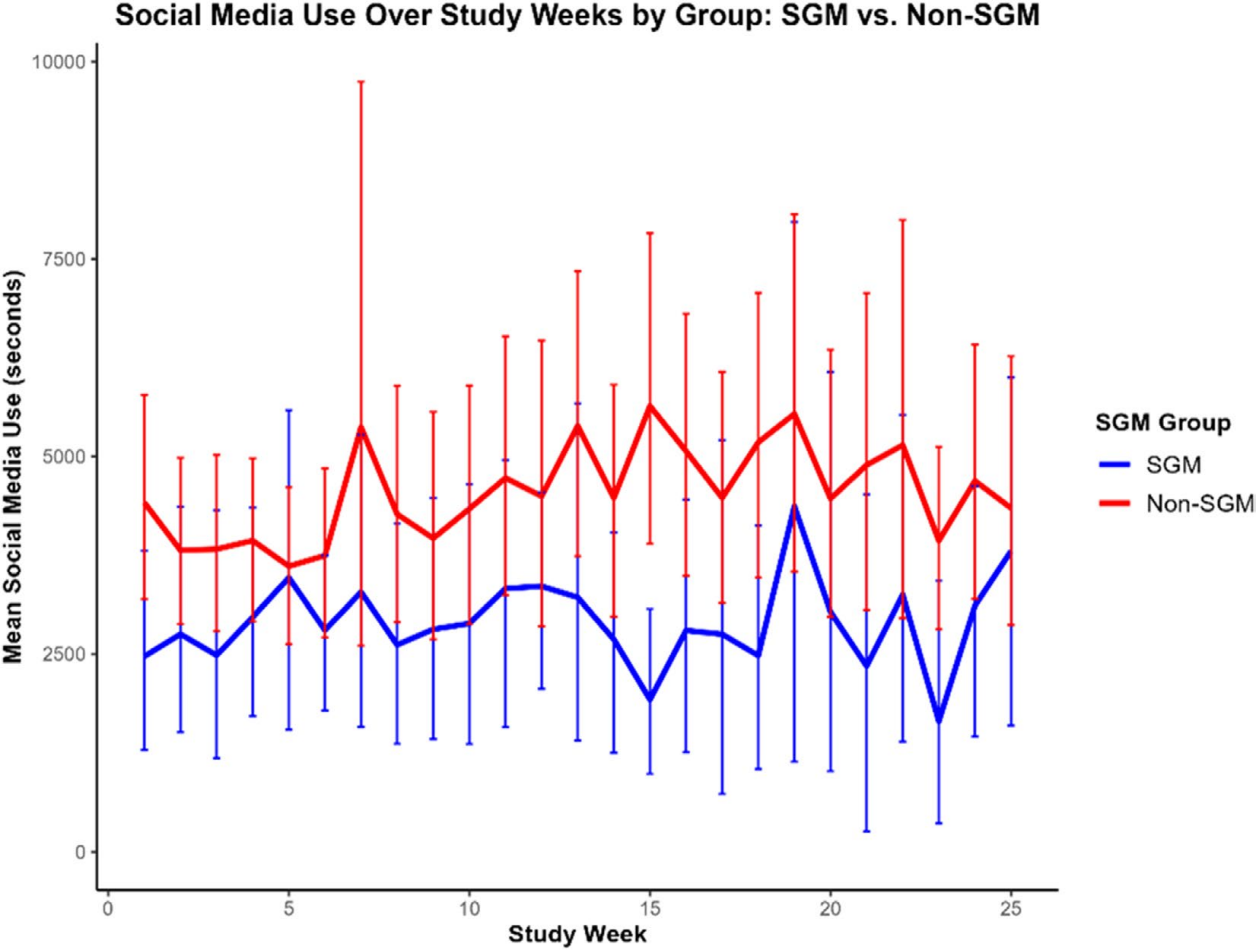
Analytical framework and decision rules for passive smartphone monitoring. The figure illustrates the full pipeline from passive data acquisition through data processing, feature derivation, dataset construction, and longitudinal modeling.

To support replication, the passive monitoring framework used in this study followed a standardized, modular pipeline consisting of four stages: (1) app‐based passive data acquisition, (2) temporal aggregation and cleaning of raw event‐level data, (3) derivation of theory‐informed behavioral features (screen use and phone‐inactivity‐based sleep proxies), and (4) longitudinal modeling using mixed‐effects regression. Each stage relied on predefined rules and thresholds, enabling reproducibility across samples and settings using comparable passive sensing platforms. For engagement purposes, weekly data checks took place to ensure all passive data was adequately collected. Additionally, participant questionnaires were monitored and troubleshooted if the participant failed to respond to the assessments. Research assistants were also in touch with both parents and adolescents if there were any technical issues with the app itself (e.g., app disconnection, bug fixes). A schematic representation of the analytical framework, including key processing steps and decision rules, is presented in Figure 1.

### Data analysis

All analyses were conducted using R (version 4.4).

#### Aim 1: Feasibility and acceptability of passive smartphone monitoring

To evaluate feasibility, we computed descriptive statistics to summarize data coverage, missingness, and key smartphone‐derived variables, including total screen time, social media use, and sleep duration. These analyses were used to assess the extent to which participants contributed usable passive sensing data over the study period and to characterize the quality and completeness of the data.

#### Aim 2: Longitudinal differences in smartphone‐derived behavioral features

To examine longitudinal differences across demographic groups, we estimated two‐level linear mixed‐effects regression models (LMMs) with random intercepts for participants using the lme4 package with Restricted Maximum Likelihood (REML). Random‐intercept models were selected to capture between‐person variability in baseline levels while maintaining parsimony and focusing on group‐average longitudinal trajectories. At Level 1 (within‐person), repeated weekly observations of total screen time, social media use, and sleep duration were modeled as a function of study week. At Level 2 (between‐person), participants were grouped according to minority status. Significance tests for fixed effects were computed using *t*‐tests with Satterthwaite's approximation of degrees of freedom.

#### Aim 2a: SGM group differences

To examine differences between sexual and gender minority (SGM) and non‐SGM adolescents, three separate LMMs were estimated, predicting total screen time, social media use, and sleep duration. Each model included fixed effects for study week, SGM status, and their interaction.

#### Aim 2b: Immigrant‐origin group differences

To examine differences between immigrant‐origin and non‐immigrant‐origin adolescents, three parallel LMMs were estimated for total screen time, social media use, and sleep duration. Each model included fixed effects for study week, immigrant‐origin status, and their interaction.

#### Design Sensitivity

To assess the sensitivity of the multilevel design, we estimated the minimum detectable effect size (MDES) for each fixed effect term in the linear mixed‐effects models. MDES values were derived from model‐based standard errors and degrees of freedom, assuming *α* = .05 and 80% power.

### Ethical approval

The study was conducted in accordance with the Declaration of Helsinki and approved by the Institutional Review Board of Rabin Medical Center (approval no. 0433‐20, December 10, 2020) and by the Ethical Review Board of the Faculty of Social Welfare and Health Sciences at the University of Haifa (approval no. 221/24, June 23, 2024).

## RESULTS

### Descriptives

The study included 99 high‐risk participants. The mean age was 14.21 years (SD = 1.8, range = 11–18). Demographic data were available for 94 participants (94.9%), as five participants (5.1%) did not provide their demographic information. As shown in Table 1, most participants identified as female (61.7%, *n* = 58), followed by male (35.1%, *n* = 33), and 3.2% (n = 3) identified as other. Most participants identified as cisgender heterosexual (87.2%, *n* = 82).

**TABLE 1:** Participant demographic characteristics.

Socio‐demographic descriptives (*n* = 94)	*n*	%
*Gender identity*		
Female	58	61.7
Male	33	35.1
Transgender female‐to‐male	5	5.3
Transgender male‐to‐female	1	1.1
Other	3	3.2
*Sexual orientation*		
Cisgender heterosexual	82	87.2
Lesbian	3	3.2
Gay	2	2.1
Bisexual	1	1.1
*Immigration (n = 70)*		
Non‐immigrant‐Origin	51	72.9
Immigrant‐Origin	19	27.1
*Religious affiliation (n = 51)*		
Secular	30	58.8
Traditional	10	19.6
Religious	10	19.6
Ultra‐orthodox	1	2.0
*Socioeconomic status (n = 72)*		
Very good	11	15.3
Good	15	20.9
Average	36	50.0
Not so good	8	11.1
Not good at all	2	2.8
*Parental employment (n = 70)*		
Employed	49	70.0
Self‐employed	11	15.7
Unemployed	10	14.3

*Note*: Immigrant‐origin status was determined by the parental country of birth.

For the study analysis, participants were categorized into two groups: non‐SGM (cisgender heterosexual) and SGM (all other identities). Immigration data was available for 70 participants. We operationalized immigrant‐origin status based on parental country of birth: participants were classified as immigrant‐origin if at least one parent was born outside the country; otherwise, they were classified as non‐immigrant‐origin. The classification of “immigration” is complex and varies across studies (Stevens & Vollebergh, 2008). However, given the demographic characteristics and context of the country where this research was conducted, this approach was deemed the most appropriate to ensure the inclusion of minority participants. The majority of participants were categorized as non‐immigrant‐origin based on this criterion (72.9%, *n* = 51), while 27.1% (*n* = 19) were categorized as immigrant‐origin.

### Aim 1: Feasibility and acceptability for passive smartphone monitoring

The raw data set initially contained 325,825 rows, where each row represented one participant‐hour on a specific date. To ensure valid estimates of sleep onset and wake‐up times, we applied additional data‐cleaning rules. Specifically, nights were excluded when the derived wake time occurred before sleep onset or when the wake time was erroneously assigned to the previous day. Out of approximately 16,200 nights, about 5800 were removed based on these criteria (36%), leaving about 10,400 valid nights. We limited the dataset to a maximum of 25 weeks/participant. The final analytic dataset comprised approximately 1500 participant‐weeks across 99 participants.

Of the 99 recruited participants, 60% (*n* = 60) completed the 6 months follow‐up period, while 40% (*n* = 39) did not complete the entire follow‐up for various reasons, including switching from Android to iPhone (incompatible with the iFeel app), undue burden of participation in active weekly digital self‐report surveys and follow‐up assessments, or any other type of parental or participant reluctance to continue in the study. Data collected before the participants' dropout were retained and included in the analyses.

Although data collection was scheduled for up to 25 weeks, no participant contributed data for all 25 weeks. Participants had intermittent missing weeks due to technical issues, device changes, or temporary disengagement. On average, participants contributed approximately 18 weeks with available passive smartphone data (e.g., screen‐use data). After applying sleep‐specific validity criteria, the number of weeks contributing usable phone‐inactivity‐based proxy sleep indicators was lower, with participants contributing an average of 11.9 valid weeks (range = 1–18). Mean numbers of sleep‐valid weeks were 14.6 among SGM participants and 11.8 among non‐SGM participants, and 13.6 among immigrant‐origin participants compared with 11.3 among non‐immigrant participants. These differences were modest in magnitude and did not suggest pronounced differential missingness across demographic subgroups. Design sensitivity analyses were based on the total number of weeks with available passive data, whereas sleep analyses were restricted to weeks meeting sleep‐proxy validity thresholds. Such levels of missingness are consistent with prior smartphone‐based sensing research (Bloom et al., 2024), where data gaps frequently arise from technical limitations, device changes, or participant disengagement. While missingness was substantial, it does not necessarily indicate systematic bias, particularly in the context of long‐term passive monitoring in high‐risk adolescent samples.

As shown in Table 2, the mean total screen time per participant was 14,249 s (SD = 6435, range = 3190–32,691), equivalent to approximately 4 h/day. Average social media usage time was 3431 s (SD = 3735, range = 0–24,049) per participant, equivalent to roughly 1 h/day. Average nightly sleep duration was 8.70 h (SD = 1.90, range = 5.24–13.00). On average, participants contributed 59.6 weekdays (Sunday–Thursday) and 23.8 weekend days (Friday–Saturday) of usable passive sensing data.

**TABLE 2:** Descriptive statistics for passive smartphone monitoring and sleep variables.

Variable	M	SD	Range	Equivalent
Total screen time (s/day)	14,249	6435	3190–32,691	4 h/day
Social media use (s/day)	3431	3735	0–24,049	1 h/day
Nightly sleep duration (h)	8.70	1.90	5.24–13.00	–

### Aim 2a: longitudinal differences in smartphone‐derived behavioral features among SGM adolescents

Table 3 summarizes the results from two‐level linear mixed‐effects models examining group differences in weekly smartphone‐derived metrics. Models included fixed effects of study week, minority status (SGM vs. non‐SGM; immigrant‐origin vs. non‐immigrant‐origin), and their interaction, with random intercepts for participants.

**TABLE 3:** Passive Smartphone Use across Diverse Minority Populations

Group	Outcome	Predictors	Estimates	SE	*p*
SGM	Total Screen Time	Intercept	15,447.219	943.722	< .001
		Study Week	−18.209	24.125	.451
		SGM vs. Non‐SGM	−34.451	2,624.203	.990
		Study Week × SGM	33.709	60.531	.578
	Social Media Use	Intercept	4,043.707	578.467	< .001
		Study Week	18.877	15.571	.226
		SGM vs. Non‐SGM	−1,273.055	1,606.768	.430
		Study Week × SGM	−11.324	39.100	.772
	Sleep Duration	Intercept	8.553	0.243	< .001
		Study Week	0.009	0.009	.305
		SGM vs. Non‐SGM	−0.484	0.666	.469
		Study Week × SGM	0.012	0.021	.576
Immigrant‐Origin^a^	Total Screen Time	Intercept	15,516.760	2,021.962	< .001
		Study Week	−73.431	47.487	.122
		Non‐Immigrant‐Origin vs. Immigrant‐Origin	368.088	2,386.910	.878
		Study Week × Non‐Immigrant‐Origin	94.689	57.507	.100
	Social Media Use	Intercept	3,172.700	1,242.220	.013
		Study Week	19.268	30.145	.523
		Non‐Immigrant‐Origin vs. Immigrant‐Origin	1,074.622	1,466.570	.466
		Study Week × Non‐Immigrant‐Origin	−4.659	36.496	.898
	Sleep Duration	Intercept	7.746	0.483	< .001
		Study Week	0.020	0.017	.237
		Non‐Immigrant‐Origin vs. Immigrant‐Origin	1.403	0.570	.**016**
		Study Week × Non‐Immigrant‐Origin	−0.042	0.020	.**038**

*Note*. Bold values indicate statistically significant predictors (*p* < .05).

Abbreviation: SGM, sexual and gender minority.

^a^ Origin status was determined by the parental country of birth.

#### Total smartphone use

There were no significant longitudinal differences in total screen time by SGM status. Study week was not significantly associated with changes in screen time (*b* = −18.21, SE = 24.13, *t* = −0.75, *p* = .451, *β* = −0.01), and neither the main effect of SGM status (*b* = −34.45, SE = 2624.20, t < −0.01, *p* = .990, *β* = 0.04) nor the interaction with study week (*b* = 33.71, SE = 60.53, *t* = 0.56, *p* = .578, *β* = 0.03) reached significance. The model predictors explained negligible variance in screen time (marginal *R* ^2^ = .000; conditional *R* ^2^ = .679). Random effects indicated substantial between‐person variability, with *τ*₀₀ = 60,391,546.69, *σ* ^2^ = 28,575,917.44, and ICC = .68.

#### Social media use

**FIGURE 2:**
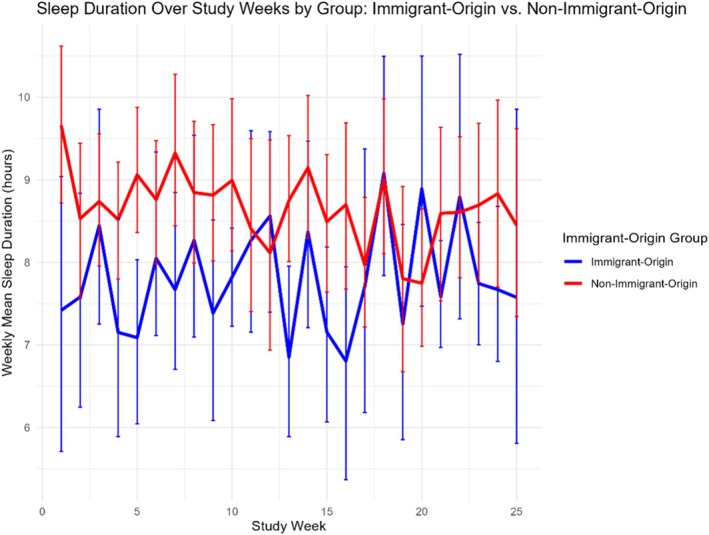
Mean social media use (in seconds) across 25 study weeks among SGM and non‐SGM adolescents. Although SGM adolescents showed slightly lower average social media use than their non‐SGM peers, longitudinal differences were not statistically significant.

Analyses revealed no significant differences in social media use between SGM and non‐SGM adolescents. Study week (*b* = 18.88, SE = 15.57, *t* = 1.21, *p* = .226, *β* = 0.02), SGM status (*b* = −1273.06, SE = 1606.77, *t* = −0.79, *p* = .430, *β* = −0.24), and their interaction (*b* = −11.32, SE = 39.10, *t* = −0.29, *p* = .772, *β* = −0.01) were nonsignificant. The model predictors explained a negligible proportion of variance (marginal *R* ^2^ = .008; conditional *R* ^2^ = .655). Random effects indicated substantial between‐person variability, with *τ*₀₀ = 22,395,641.68, *σ* ^2^ = 11,930,476.41, and ICC = .65. As shown in Figure 2, both SGM and non‐SGM adolescents demonstrated comparable and relatively stable social media use across the 25 study weeks.

#### Sleep duration

No significant differences in sleep duration were observed by SGM status. Study week (*b* = 0.009, SE = 0.009, *t* = 1.03, *p* = .305, *β* = 0.03), SGM status (*b* = −0.484, SE = 0.666, *t* = −0.73, *p* = .469, *β* = −0.14), and the interaction (*b* = 0.012, SE = 0.021, *t* = 0.56, *p* = .576, *β* = 0.03) were all nonsignificant. The model predictors explained negligible variance in sleep duration (marginal *R* ^2^ = .003; conditional *R* ^2^ = .506). Random effects indicated substantial between‐person variability, with *τ*₀₀ = 3.55, *σ* ^2^ = 3.49, and ICC = .50.

### Aim 2b: longitudinal differences in smartphone‐derived behavioral features among immigrant‐origin adolescents

#### Total smartphone use

No significant longitudinal differences were observed in total screen time between immigrant‐origin and non‐immigrant‐origin adolescents. Study week (*b* = −73.43, SE = 47.49, *t* = −1.55, *p* = .122, *β* = −0.05), immigrant‐origin status (*b* = 368.09, SE = 2386.91, *t* = 0.15, *p* = .878, *β* = 0.15), and their interaction (*b* = 94.69, SE = 57.51, *t* = 1.65, *p* = .100, *β* = 0.07) were nonsignificant. The model predictors explained negligible variance (marginal *R* ^2^ = .005; conditional *R* ^2^ = .672). Random effects indicated substantial between‐person variability, with *τ*₀₀ = 70,347,173.15, *σ* ^2^ = 34,619,028.10, and ICC = .67.

#### Social media use

There were no significant effects of study week (*b* = 19.27, SE = 30.15, *t* = 0.64, *p* = .523, *β* = 0.02), immigrant‐origin status (*b* = 1074.62, SE = 1466.57, *t* = 0.73, *p* = .466, *β* = 0.16), or the interaction (*b* = −4.66, SE = 36.50, *t* = −0.13, *p* = .898, *β* = −0.01) on social media use. The model predictors explained negligible variance (marginal *R* ^2^ = .006; conditional *R* ^2^ = .656). Random effects indicated substantial between‐person variability, with *τ*₀₀ = 26,364,340.08, *σ* ^2^ = 13,954,849.62, and ICC = .65.

#### Sleep duration

**FIGURE 3:**
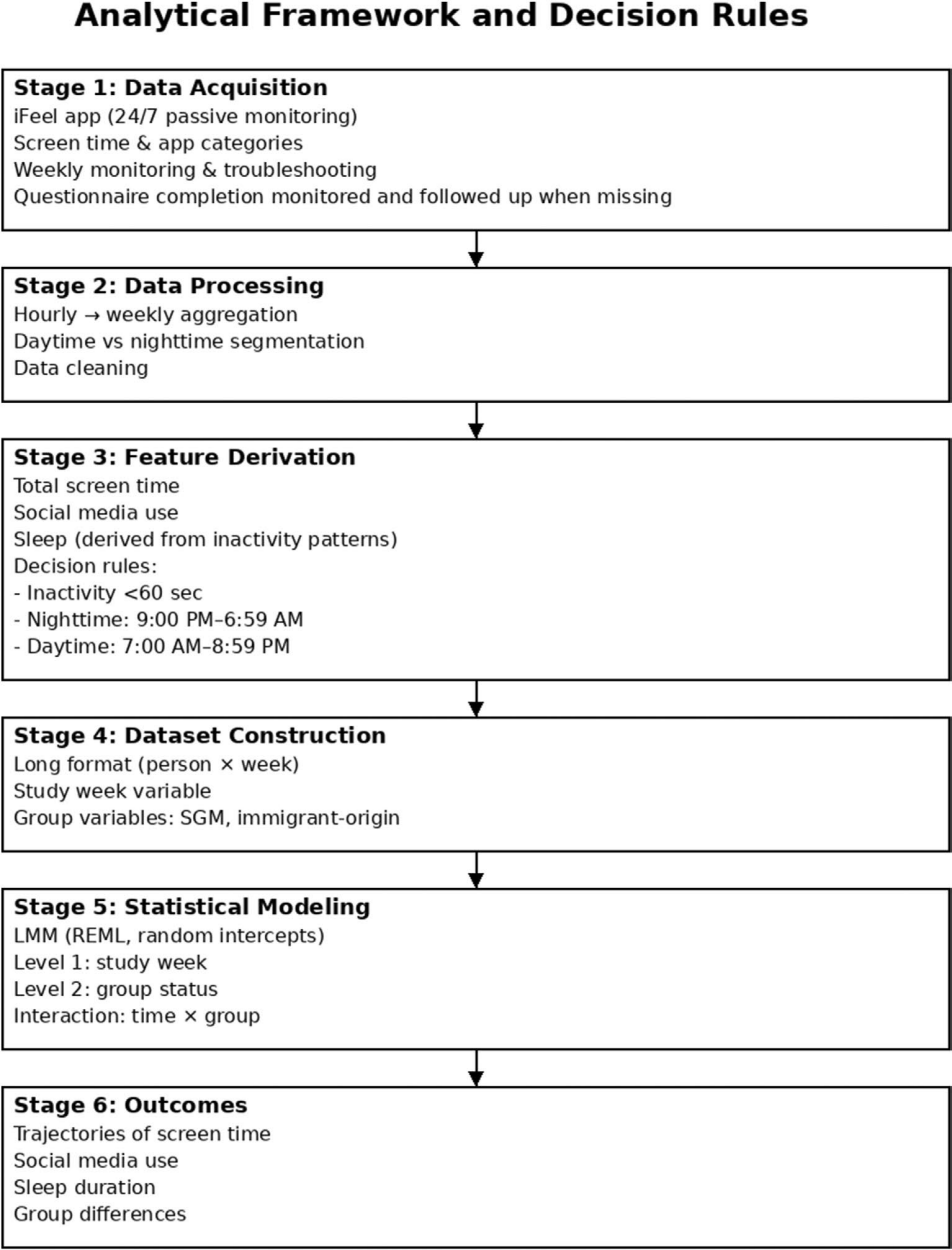
Mean weekly sleep duration (in hours) across 25 study weeks among immigrant‐origin and non‐immigrant‐origin adolescents. Immigrant‐origin adolescents exhibited consistently shorter but relatively stable sleep durations, whereas non‐immigrant‐origin adolescents showed longer initial sleep durations followed by a gradual decline over time, reflecting a significant interaction between study week and immigrant‐origin status.

Analyses revealed significant differences in sleep duration. Immigrant‐origin adolescents had shorter nightly sleep duration compared to non‐immigrant‐origin adolescents (*b* = 1.40, SE = 0.57, *t* = 2.46, *p* = .016, *β* = 0.35, 95% CI [0.27, 2.54]). This effect was qualified by a significant interaction between study week and immigrant‐origin status (*b* = −0.042, SE = 0.020, *t* = −2.07, *p* = .039, *β* = −0.11, 95% CI [−0.081, −0.002]), indicating that sleep duration among immigrant‐origin adolescents remained more stable across weeks compared to non‐immigrant‐origin adolescents, whose sleep declined more sharply over time. The main effect of the study week was not significant (*b* = 0.020, SE = 0.017, *t* = 1.18, *p* = .237, *β* = 0.05). The model predictors explained a modest proportion of variance (marginal *R* ^2^ = .027; conditional *R* ^2^ = .493). Random effects indicated substantial between‐person variability, with *τ*₀₀ = 3.49, *σ* ^2^ = 3.79, and ICC = .48. As shown in Figure 3, immigrant‐origin adolescents exhibited consistently shorter but relatively stable sleep durations across the study period, whereas non‐immigrant‐origin adolescents demonstrated longer initial sleep durations followed by a gradual decline over time.

### Design sensitivity

Design‐sensitivity analyses were conducted to evaluate the precision of the longitudinal multilevel models. Based on the observed sample size (*N* = 99 adolescents) and an average of 17–18.5 weekly assessments per participant (≈1700 total observations), power analyses indicated that the study achieved approximately 80% power at *α* = .05 to detect small‐to‐moderate time × minority interaction effects. For models predicting total screen time and social media use, the minimum detectable effect size (MDES) ranged from approximately 1.5–3 min/day, whereas for sleep‐duration models, the MDES corresponded to approximately 0.05–0.07 h/day (i.e., ~3–4 min/day, averaged across the week). These estimates indicate that the design was sufficiently sensitive to detect subtle within‐person temporal changes, particularly in sleep duration. Random‐effects estimates revealed substantial between‐person variability across outcomes, with intraclass correlation coefficients (ICC) ranging from .48 to .68, supporting the inclusion of random intercepts in the models. Although the observed group differences were not statistically significant, the sensitivity analysis suggests that similar effects might reach significance in studies with larger samples or reduced within‐person variability.

## DISCUSSION

The current study aimed to provide a replicable framework for collecting and analyzing passive smartphone data among adolescents. The approach showed strong feasibility and engagement, with most participants contributing multiple weeks of usable data. High‐quality data were obtained for behavioral metrics: screen time, social media activity time, and sleep duration. While no longitudinal group differences by SGM status were observed, immigrant‐origin adolescents displayed slightly shorter but more stable sleep across the study period. These findings support the viability of continuous passive monitoring in adolescent populations and its potential to extend research efforts to the understanding of context‐sensitive real‐world mental health challenges in adolescents.

### Feasibility, engagement and accessibility

Despite the challenges of sustaining longitudinal digital data collection (Bloom et al., 2024), most participants provided valid data for an average of 12 weeks. This high level of adherence likely reflects the proactive monitoring procedures used in this study, including weekly data checks, technical troubleshooting, and direct participant support. Such active engagement aligns with previous findings that regular researcher contact improves retention in smartphone‐based studies (Clark et al., 2025). These efforts likely minimized data loss and fostered trust, contributing to the overall feasibility of the approach. The passive monitoring system also demonstrated high sensitivity in detecting within‐person changes in digital behavior, indicating that meaningful day‐to‐day variations could be captured without participant effort. This combination of high feasibility, sustained engagement, and sensitive behavioral detection underscores the potential of passive smartphone monitoring as a scalable and accessible tool for longitudinal research with adolescents.

Retention in longitudinal research is particularly challenging among *“traditionally difficult‐to‐follow populations”*, such as individuals with severe mental illness (Gibbons et al., 2010). Yet, participants in the current study maintained steady engagement throughout the monitoring period – an encouraging outcome particularly given that depressive symptoms are frequently associated with lower levels of both active and passive data contribution (Zhang et al., 2023). The sustained engagement observed in the current study may also reflect the relatively low participant burden of passive smartphone monitoring compared to active EMA protocols. While EMA is generally feasible and well accepted, repeated assessments can induce fatigue and lead to declining compliance over time (Sedano‐Capdevila et al., 2021). In contrast, passive data collection, as was utilized in the current study, required no active input from participants, likely reducing burden and contributing to the stable retention observed during the study weeks.

Beyond feasibility, the study also demonstrated the potential of passive smartphone monitoring to engage adolescents who are often underrepresented in mental health‐based research. Many of these youth face barriers to traditional psychological services and may not seek help through conventional pathways (Higgins et al., 2021). Prior work has demonstrated the feasibility of intensive smartphone‐based methods among SGM adolescents (Clark et al., 2025). Consistent with this evidence, the present findings suggest that passive smartphone monitoring can provide a low‐burden, scalable approach to engage youth who might otherwise remain outside clinical or research settings. By reducing self‐report and active participation, this method may help expand access and representation in studies examining adolescents' real‐world behavioral patterns using passive digital data.

### Comparison with normative data and ecological validity

The behavioral metrics obtained in this study closely align with established norms for adolescent smartphone use and sleep duration, supporting the ecological validity of the passive monitoring data. On average, participants spent approximately 4 h/day on their smartphones and about 1 h on social media platforms. These values are consistent with recent large‐scale surveys reporting that adolescents typically engage with their smartphones for around 5 h daily and spend about 1.5 h on social media (Alexander et al., 2024; Wang et al., 2025). Although no direct comparison group was included, these findings indicate that smartphone usage indices within this sample fell well within the range observed in normative adolescent populations.

The average nightly sleep duration of 8.7 h observed in this sample aligns with public health recommendations of 8–10 h for adolescents (The National Sleep Foundation, 2020), but appears somewhat longer than typically reported in empirical sleep studies. For instance, Lucas‐Thompson et al. (2021) found that adolescents self‐reported an average sleep duration of approximately 8.25 h and approximately 6.9 h when measured by actigraphy, whereas Lemke et al. (2025) similarly reported that adolescents experiencing suicidal ideation slept around 7 h/night. The slightly longer sleep durations in the current study likely reflect methodological differences, as sleep onset and offset were derived from passive smartphone data rather than self‐reported or actigraphic measures. Importantly, these results demonstrate that passive smartphone monitoring can capture both sleep and smartphone use in real‐world contexts, offering a feasible, low‐burden alternative to traditional assessment methods while yielding behavior estimates consistent with normative adolescent data.

### Behavioral patterns across diverse groups

The longitudinal scope of the current dataset allowed us to examine potential disparities across diverse adolescent groups. Contrary to expectations, no significant longitudinal differences emerged between SGM and non‐SGM adolescents. Within this clinical sample, SGM participants showed slightly lower social media use than their non‐SGM peers. This contrasts with findings from community samples, where SGM adolescents typically report higher engagement and greater risk for problematic social media use (Kaiser et al., 2021). This discrepancy may reflect context‐specific factors unique to adolescents experiencing STB, such as lower peer connectedness (Barzilay et al., 2019). Together, these findings highlight that digital behavior among SGM adolescents with STB may differ from that seen in community samples, emphasizing the need for context‐sensitive interpretations of digital behavior in marginalized adolescents.

In contrast, the results for immigrant‐origin adolescents revealed a distinct temporal pattern. Non‐immigrant‐origin adolescents exhibited longer sleep duration at baseline but steeper declines over time, whereas immigrant‐origin adolescents maintained shorter yet more stable sleep across the study period. This aligns with prior evidence showing that children of migrant origins often sleep less than their native‐born peers (Labree et al., 2015). Importantly, a substantial proportion of variance in sleep duration was attributable to differences between‐person, suggesting that sleep patterns are largely driven by stable individual characteristics, whereas the predictors in the model accounted for only a small proportion of variability over time. Research on sleep and smartphone behavior among immigrant‐origin adolescents remains limited, particularly within clinical samples. Thus, these findings should be considered preliminary, warranting replication in larger and more diverse cohorts to clarify how migration‐related factors shape digital engagement and sleep during adolescence.

Overall, these findings indicate that behavioral disparities among minority adolescents are dynamic rather than static. They likely reflect the intersection of cultural, contextual, and familial factors rather than identity status alone. By including both SGM and immigrant‐origin adolescents, this study broadens representation within passive sensing research and contributes to a more inclusive understanding of digital behavior among adolescents.

### Implications

This study provides a practical framework for implementing passive smartphone monitoring in contextually sensitive mental health research among adolescents. It demonstrates the technical feasibility necessary for applying this approach in clinical and community settings and, by documenting each step of data acquisition, processing, and analysis, offers a replicable methodological guide for future researchers. Replication of this framework requires a passive sensing system capable of recording timestamped screen activity at an hourly resolution, a predefined nighttime window for sleep inference, and application of standardized inactivity thresholds and aggregation rules as described in the Methods; importantly, the framework is not dependent on a specific application or proprietary platform.

Additionally, this work addresses issues of representation and equity. By including SGM and immigrant‐origin adolescents, two groups often underrepresented in digital mental health research, it broadens the populations studied using passive sensing. These methodological and sampling strategies illustrate how passive monitoring can enhance inclusivity and yield a deeper understanding of behavioral patterns among diverse adolescents. Such approaches are particularly relevant given their nuanced ecological validity and unobtrusive nature. Clinically, these methods hold promise for informing culturally sensitive, scalable systems for passively monitoring longitudinal behavioral patterns, providing a foundation for future studies examining their association with suicide risk.

### Limitations

Consistent with prior passive monitoring research (Bloom et al., 2024), the dataset contained missing data due to technical issues, data stream disruptions, phone replacements, and temporary disengagement. Second, smartphone activity was defined as any use lasting at least 60 s. This approach may not fully distinguish between active and incidental use (e.g., brief notifications) or capture wake time without any phone use. Thus, the derived sleep measures should be interpreted as phone‐inactivity‐based proxy sleep indicators of inactivity rather than verified sleep. Third, passive sensing captures only the quantity and timing of phone use, without contextual information about specific activities or emotional significance. Fourth, as the sample consisted of adolescents referred to mental health treatment and at risk for STB, findings may not generalize to other populations and settings. Fifth, it is important to note that this study took place during the COVID‐19 pandemic. Timing related to recruitment and study participation could have been influenced given the point in time. Sixth, the sample comprised 12 SGM adolescents and 82 cisgender heterosexual adolescents. This sample does not represent an adequately diversified group, and therefore findings should be interpreted with this in mind. Lastly, the immigration‐origin group was defined by parental birthplace, which simplifies the complex sociocultural dimensions of migration and identity. This is important to note as the definition of immigrant‐origin status varies across studies (Stevens & Vollebergh, 2008).

### Future studies

Given that participants contributed an average of 12 weeks of usable data, shorter monitoring periods may improve engagement. Establishing dedicated technical support and maintaining consistent participant contact could further enhance data compliance. Future research should move beyond duration‐based metrics to explore the qualitative nature of digital engagement, such as what adolescents are doing on their phones, not just how long they use them. Investigating mediating mechanisms (e.g., whether smartphone use influences STB through sleep disturbances and social isolation) will be valuable for clarifying pathways linking digital behavior and suicide risk and identifying more targeted interventions. Additionally, examining day‐to‐day and weekday‐weekend variations in use may help us understand behavioral patterns linked to mood fluctuations and social connections.

## CONCLUSION

To our knowledge, this study is among the first to outline a methodological framework for implementing passive smartphone monitoring in adolescents. By including SGM and children of immigrant parents, it offers a rare view of real‐world behavioral patterns among groups often underrepresented in mental‐health research, which may be utilized in future studies of adolescents from other minority groups. This work contributes to a growing body of evidence supporting passive sensing as an inclusive and scalable approach for capturing daily functioning and identifying dynamic risk states in vulnerable populations. Future studies should build on this framework with larger and more diverse samples to advance understanding of digital behavior and mental health among adolescents.

## AUTHOR CONTRIBUTIONS

**Noa Benaroya‐Milshtein:** Supervision; writing – review and editing; resources. **Shai Fine:** Writing – review and editing; supervision; resources; software; methodology; funding acquisition. **Talia Friedman:** Resources; data curation; software; methodology. **Lior Carmi:** Software; data curation; resources; methodology. **Maya Stemmer:** Resources; data curation; software; methodology. **Oren Shahnovsky:** Conceptualization; investigation; funding acquisition; writing – original draft; methodology; visualization; formal analysis; data curation. **Daniella Ekstein:** Writing – original draft; writing – review and editing; methodology. **Shira Barzilay:** Writing – review and editing; funding acquisition; formal analysis; project administration; supervision; resources; validation; methodology; conceptualization.

## FUNDING INFORMATION

This work was supported by the Graduate Studies Authority and the Bloom School of the University of Haifa, the Bishvil Hahayim (R.A.) Association, and the Data Science Institute of Reichman University. Partial support was also provided by the Lior Zfati Center for Suicide and Mental Pain Research and by the Israel Ministry of Science and Technology (grant number 3‐15289). The content is solely the responsibility of the authors and does not necessarily represent the official views of the funding sources.

## CONFLICT OF INTEREST STATEMENT

No potential conflict of interest was reported by the author(s).

## ETHICS STATEMENT

The study was conducted in accordance with the Declaration of Helsinki and approved by the Institutional Review Board of Rabin Medical Center (approval no. 0433‐20, December 10, 2020) and by the Ethical Review Board of the Faculty of Social Welfare and Health Sciences at the University of Haifa (approval no. 221/24, June 23, 2024).

## PATIENT CONSENT STATEMENT

Written parental consent and child assent (for participants aged 16 years and older) were obtained from all participants prior to enrollment.

## Supporting information


**Appendix S1.** Application categories captured by the passive smartphone monitoring system.

## Data Availability

The data that support the findings of this study are available from the corresponding author upon reasonable request.
