# Supplementary material for: Understanding sleep and smartphone use in diverse adolescents through passive digital monitoring
Source: J Res Adolesc. 2026 May 6;36:e70192. doi: 10.1111/jora.70192 (PMC13147491; doi:10.1111/jora.70192)
Supplement: Supplementary file 1 — Appendix S1. Application categories captured by the passive smartphone monitoring system. [file JORA-36-0-s001.docx]

**Supplementary Appendix S1.** *Application Categories Captured by the Passive Smartphone Monitoring System*

The passive smartphone monitoring system categorized application usage based on the predefined Google Play application taxonomy. The following application categories were included:

1. Art & Design
2. Auto & Vehicles
3. Books & Reference
4. Browsers
5. Business
6. Comics
7. Communication
8. Dating
9. Education
10. Entertainment
11. Events
12. Finance
13. Food & Drink
14. Games
15. Health & Fitness
16. Lifestyle
17. Maps & Navigation
18. Medical
19. Music & Audio
20. News & Magazines
21. Parenting
22. Personalization
23. Photography
24. Shopping

*Note. Application categories reflect the Google Play Store classification at the time of data collection. Time spent within each category was aggregated at the hourly level and subsequently summarized into weekly averages for analysis.*
